# Inferring epidemiologic dynamics from viral evolution: 2014–2015 Eurasian/North American highly pathogenic avian influenza viruses exceed transmission threshold, *R*_0_ = 1, in wild birds and poultry in North America

**DOI:** 10.1111/eva.12576

**Published:** 2017-12-01

**Authors:** Daniel A. Grear, Jeffrey S. Hall, Robert J. Dusek, Hon S. Ip

**Affiliations:** ^1^ United States Geological Survey National Wildlife Health Center Madison WI USA

**Keywords:** basic pathogen reproductive number, highly pathogenic avian influenza, phylodynamics, phylogeny, poultry, *R*_0_, whole‐genome sequence, wild birds

## Abstract

Highly pathogenic avian influenza virus (HPAIV) is a multihost pathogen with lineages that pose health risks for domestic birds, wild birds, and humans. One mechanism of intercontinental HPAIV spread is through wild bird reservoirs, and wild birds were the likely sources of a Eurasian (EA) lineage HPAIV into North America in 2014. The introduction resulted in several reassortment events with North American (NA) lineage low‐pathogenic avian influenza viruses and the reassortant EA/NA H5N2 went on to cause one of the largest HPAIV poultry outbreaks in North America. We evaluated three hypotheses about novel HPAIV introduced into wild and domestic bird hosts: (i) transmission of novel HPAIVs in wild birds was restricted by mechanisms associated with highly pathogenic phenotypes; (ii) the HPAIV poultry outbreak was not self‐sustaining and required viral input from wild birds; and (iii) reassortment of the EA H5N8 generated reassortant EA/NA AIVs with a fitness advantage over fully Eurasian lineages in North American wild birds. We used a time‐rooted phylodynamic model that explicitly incorporated viral population dynamics with evolutionary dynamics to estimate the basic reproductive number (*R*_0_) and viral migration among host types in domestic and wild birds, as well as between the EA H5N8 and EA/NA H5N2 in wild birds. We did not find evidence to support hypothesis (i) or (ii) as our estimates of the transmission parameters suggested that the HPAIV outbreak met or exceeded the threshold for persistence in wild birds (*R*_0_ > 1) and poultry (*R*_0_ ≈ 1) with minimal estimated transmission among host types. There was also no evidence to support hypothesis (iii) because *R*_0_ values were similar among EA H5N8 and EA/NA H5N2 in wild birds. Our results suggest that this novel HPAIV and reassortments did not encounter any transmission barriers sufficient to prevent persistence when introduced to wild or domestic birds.

## 1 INTRODUCTION

In the late autumn of 2014, a highly pathogenic avian influenza virus (HPAIV) was discovered in wild birds and poultry in southwestern Canada and northwestern United States (USA). This HPAIV H5N8 viral lineage was widely distributed in Asia, emerging in Europe, and likely transported to North America by migrating wild birds across the Bering Strait (Lee et al., 2015; Lycett et al., 2016). The H5N8 virus reassorted with indigenous North American low‐pathogenic influenza viruses (LPAIV), creating mixed Eurasian/North American (EA/NA) lineage H5N1 and H5N2 HPAI viruses. These viruses, particularly the EA/NA H5N2 subtype, spread through the northwestern USA and into the central USA where the EA/NA HPAIV H5N2 virus infected poultry operations leading to one of the worst agricultural epizootic in US history. From the initial introduction to the end of the outbreak in June 2015, 50 million domestic poultry were dead from viral infection or depopulation efforts to control the epidemic (Greene, 2015). The staggering economic consequences to the poultry industry, local labor forces, communities, and government agencies eventually totaled more than US $3 billion (Greene, 2015).

The high rates of evolution in RNA viruses like AIV, increasing computational power, and wide availability of genomic data from high‐consequence pathogens have resulted in conceptual advances that integrate evolutionary and epidemiological dynamics (Pybus, Fraser, & Rambaut, 2013). The emerging field of phylodynamics has extended study of viral population demographics and pathogen evolution to examine transmission dynamics related to discrete host traits such as species, geography, or host phenotype (De Maio, Wu, O'Reilly, & Wilson, 2015; Kamath et al., 2016; Kuhnert, Stadler, Vaughan, & Drummond, 2016; Stadler & Bonhoeffer, 2013). These advances are particularly useful for the study of wildlife pathogens because traditional epidemiological methods are often limited by the difficulty of sampling appropriate host information (Blanchong, Robinson, Samuel, & Foster, 2016). In this study, we used a joint evolutionary‐epidemiological model that estimated transmission dynamics within and between a subdivided host population, referred to as a birth–death multitype model (bdmm; Kuhnert et al., 2016). Notably, these methods provide an estimate of *R*_0_, the basic reproductive number of a pathogen, within host types, as well as migration rates of the pathogen between host types. This approach allowed us to frame questions about fundamental epidemiologic and evolutionary processes of the 2014–2015 North American HPAIV outbreak to generate robust conclusions about the epidemiologic dynamics in a multihost system.

The objectives of this analysis were to address hypotheses about viral dynamics in wild birds and poultry, as well as the consequences of reassortment between North American and Eurasian avian influenza viral segments for transmission in wild birds. We focused on the Eurasian hemagglutinin (HA) genome segment nucleotide sequences that were part of clade 2.3.4.4 HPAIVs (Smith & Donis, 2015), as well as the other Eurasian genome segments that persisted throughout the North American outbreak in 2014–2015, polymerase subunit (PB2) and the matrix protein (M), to make inference to postdetection dynamics in North America. Specifically, we generated quantitative evidence to evaluate the hypotheses that (i) persistence of HPAIVs in wild hosts is unlikely owing to unobserved mechanisms that suppress transmission capacity of highly pathogenic phenotypes (Krauss et al., 2016) by testing the prediction that *R*_0_ < 1 in wild bird hosts; (ii) HPAIV poultry epidemics are not self‐sustaining and require viral input from wild birds by testing the prediction that *R*_0_ < 1 in domestic bird hosts; and (iii) that reassortment of the fully Eurasian H5N8 with North American LPAIVs to generate the EA/NA H5N2 provided a fitness advantage to the reassortant AIV by testing the prediction that *R*_0_ of EA/NA H5N2 > *R*_0_ of EA H5N8 in North American wild bird hosts.

## 2 METHODS

All data were obtained from the National Institute for Allergy and Infectious Diseases Influenza Research Database (IRD) through the web site at http://www.fludb.org (Squires et al., 2012; accessed 26 October, 2016). We obtained nucleotide sequences of complete segments of the HA segment isolated from avian species in Canada and the USA that were part of clade 2.3.4.4 HPAIVs (Smith & Donis, 2015). Next, we obtained full sequences of the M segment and viral polymerase complex PB2 segments isolated from the same host, identified via the strain name.

Nucleotide sequences of each segment were aligned using the multiple sequence alignment algorithm implemented in the R package DECIPHER (r function AlignSeqs; Wright, 2015, 2016). Following initial alignment, areas of the multiple alignments with information content <0.5 bits and greater than 20% of sequences containing gaps were masked using moving averages of 10 nucleotides (r function MaskAlignment; Wright, 2016). Final sequence alignments were near full length for each segment with masked regions occurring only at the 3′ and 5′ ends (HA 1744 nucleotides, PB2 2280 nucleotides, M 987 nucleotides).

We modeled the phylodynamics of the clade 2.3.4.4 HA, PB2, and M segments using a multitype birth–death process on a time‐rooted phylogenetic tree implemented in a Bayesian framework to make inference on the basic reproduction number (*R*_0_) of each segment during the sampling of the outbreak and viral migration between host types using two nested analyses (Kuhnert et al., 2016). The isolates (tips on the phylogenetic tree) were annotated with strain name defined in the IRD, sample collection date, host type (wild bird or poultry/domestic bird), host species, state or province of collection, viral subtype (defined by HA and Neuraminidase [N] combination), and sequence accession identifier (Table S1). This family of birth–death phylodynamic models integrated uncertainty of the phylogeny of isolated sequences with an epidemiological model analogous to a compartmental model (Kuhnert, Stadler, Vaughan, & Drummond, 2014). We used the general time‐reversible (GTR) +Γ_4_ + I model with a relaxed log‐normal molecular clock to estimate the phylogeny (Chen & Holmes, 2006). We used vague informative priors for the mean clock rate based on previously estimated rates for each segment (Chen & Holmes, 2006; Tables S2–S7). Distributions for specified priors along with their posterior estimates and convergence diagnostic statistics are presented in Tables S2–S7.

The first analysis used all isolated sequences from the HA, PB2, and M segments, separately, to analyze the transmission dynamics among host types defined as wild bird or poultry/domestic bird. Owing to the short time span of the outbreak (earliest isolate collection 2 December 2014; last isolate: 1 June 2015), we estimated constant transmission model rates for the entire time period with no sampling before or after the earlier and latest isolates, respectively. We assumed that once sampled, that virus was removed from the population because wild bird sampling was from a mortality event or hunter harvest (Bevins et al., 2016) and infected poultry operations were subject to high biosecurity and depopulation once infection was detected (the point in time where the isolate was obtained; USDA APHIS 2015). For epidemiological parameters, we used the multitype birth–death model and parameter notation of Kuhnert et al. (2016). The primary epidemiological processes of interest were the basic reproduction number within each host type (*R*_0_poultry_ and *R*_0_wild_), the rate of becoming noninfectious (δ; assumed to be the same per host type), the migration rate of viral lineages among subtypes (*m*_poultry_to_wild_ and *m*_wild_to_poultry_), and the probability of sampling a viral lineage per subtype (ψ_poultry_ and ψ_wild_). Using Bayesian methods, we jointly estimated these fundamental epidemiological parameters with the evolutionary model governing the nucleotide changes and were able to infer a time‐rooted phylogeny, estimate ancestral host types of common ancestors, and estimate the relative contribution of viral transmission within host types to viral migration between host types (Kuhnert et al., 2016). We used this analysis to address the first two hypotheses by testing the prediction that *R*_0_wild_ < 1 and *R*_0_poultry_ < 1 during the 2014–2015 outbreak.

We performed a second analysis to evaluate the hypothesis that recombination of the ancestral Asian virus (EA H5N8) with a North American virus to produce the EA/NA H5N2 increased the transmission among wild bird hosts. Again, we used the multitype birth–death model and included only HA, M, and PB2 sequences isolated from wild bird hosts where there was evidence of transmission among wild birds (Lee et al., 2015). We defined this set of viral isolates as all publically available wild bird sequences collected prior to 1 February 2015. We defined the host type based on the HA and N segment subtype that was infecting the wild bird hosts (Table S1): H5N8 subtypes had a full set of Eurasian gene segments (Ip et al., 2015; Lee et al., 2015) and H5N2 was the Eurasian–North American reassortment where the PB1, NS, NP, and N segments were of North American origin and were consistent through the outbreak (Lee, Torchetti, Killian, Deliberto, & Swayne, 2017; Pasick et al., 2015). We excluded the H5N1 reassortant isolates because there were too few to make confident inferences. We used the same molecular clock model as the analysis of the full set of sequences (priors listed in Tables S2–S7) to estimate the epidemiological parameters: the basic reproduction number within each host type (*R*_0_H5N2_ and *R*_0_H5N8_), the rate of becoming noninfectious (δ; assumed to be the same per subtype), the migration rate of viral lineages among subtypes (*m*_H5N2_to_H5N8_ and *m*_H5N8_to_H5N2_), and the probability of sampling a viral lineage per subtype (ψ_H5N2_ and ψ_H5N8_). We used this analysis to address the third hypothesis by testing the prediction that *R*_0_H5N2_ > *R*_0_H5N8_ in wild birds. We used vague informative priors for the *R*_*0*_, δ, and *m* parameters. The priors for *R*_*0*_ and *m* were chosen to limit unrealistically high parameter values. The prior for δ was based on the infectious period of avian influenzas (Aldous et al., 2010) with additional uncertainty from the interpretation of host‐specific infectious periods relative to the phylodynamic model analog: lineage infectious period that may span multiple host infections. We used uninformative priors for the ψ parameters and distributions for specified priors, along with their posterior estimates and convergence diagnostic statistics are presented in Tables S2–S7.

We made inference to the phylogenetic and epidemiological parameters in a Bayesian framework using the bdmm model (Kuhnert et al., 2016; available at https://github.com/denisekuehnert/bdmm, accessed 19 September 2016) implemented using Markov chain Monte Carlo (MCMC) methods in BEAST v2.4.3 (Bouckaert et al., 2014). We ran four separate chains of 8–10 million MCMC iterations with the same priors and randomly selected starting values (Tables S2–S7). We ensured convergence of each chain by visually assessing MCMC traces for each parameter and whether effective sample sizes were sufficiently large (>200). We then performed a Gelman–Rubin diagnostic implemented in the coda package for R (Plummer, Best, Cowles, & Vines, 2006) for the MCMC chains excluding a 10% burn‐in on each parameter to ensure that scale reduction factor estimates were all <1.1 (Gelman et al., 2014). Once we confirmed convergence, we discarded 10% burn‐in from each chain, combined chains using the program Log Combiner v2.4.2 (http://beast.bio.ed.ac.uk/logcombiner), and sampled from the posterior distribution of parameters every 1,000 MCMC iterations. We reported the full set of model parameter priors, posterior estimates (mean and 95% highest posterior density interval, HPD), and convergence diagnostics for all three segments for the full wild‐poultry and nested wild bird analyses (Tables S2–S7). We focused on reporting estimated divergence times along with the epidemiological parameters for the wild‐poultry type analysis (hypotheses 1 & 2) with all the sequences and only the epidemiological parameters for the wild H5N2‐H5N8 subtype analysis on the isolate subset (hypothesis 3). In addition to the epidemiological rate parameters for the poultry and wild host type analysis (*R*_0_poultry_, *R*_0_wild_, *m*_poultry_to_wild_, *m*_wild_to_poultry_, ψ_poultry_ and ψ_wild_ *,* δ), we also calculated estimated numbers of realized transmission events from poultry to wild birds and vice versa, based on the viral migration rates (*m*_poultry_to_wild_ and *m*_wild_to_poultry_) and the relative frequencies of the ancestral host states estimated from the phylogenetic model (Kuhnert et al., 2016).

## 3 RESULTS

We analyzed 85 isolates, each with complete sequences of the HA, PB2, and M segments (Table S1) using a bdmm model that estimated time‐rooted phylogenetic and epidemiologic parameters in a Bayesian framework (Kuhnert et al., 2014). Forty sequences were isolated from domestic birds and 45 from wild birds (Table S1). Bayesian estimation of the phylogenetic and epidemiologic parameters visually converged after a 10% burn‐in and the Gelman–Rubin scale reduction factors across four MCMC chains for each parameter in each segment was <1.1 with the exception of one nucleotide transition in the M segment of the wild bird analysis (Tables S2–S7). Kuhnert et al. (2014) and Stadler, Kühnert, Bonhoeffer, and Drummond (2013) pointed out issues with correlation among the posterior estimates of transmission parameters without informative priors. Visual inspection of posterior MCMC chains and sampled posterior parameter correlation showed no evidence of parameter correlation, indicating that our choice of priors plus the data were informative enough to provide identifiable posterior estimates.

**Figure 1:**
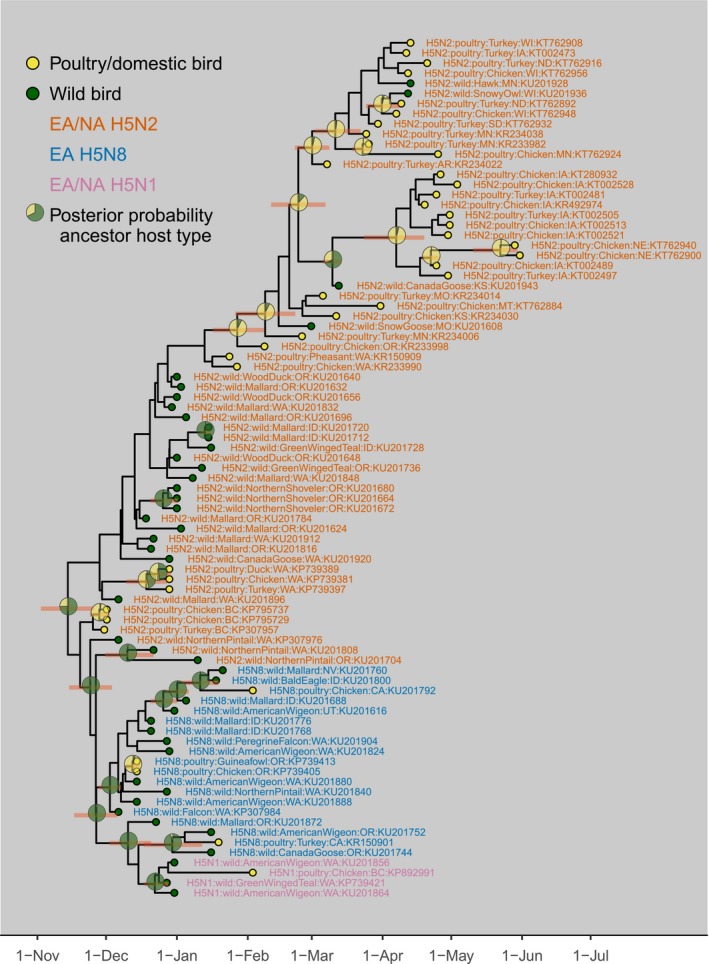
Time‐rooted maximum clade credibility phylogenetic tree of the hemagglutinin segment of highly pathogenic avian influenza viruses isolated from wild birds and poultry during the 2014–2015 outbreak in North America. Eurasian (EA) source H5N8 subtype reassorted with North American (NA) low‐pathogenic viruses to form EA/NA H5N2 and EA/NA H5N1 subtypes. Tree tip circle colors represent host types of the isolates and pie charts on internal nodes display the posterior probability of host type of common ancestor viruses at majority rule common ancestors (posterior node probability > .5). Bars represent uncertainty (95% highest posterior density intervals) of the divergence time of majority rule common ancestors

**Figure 2:**
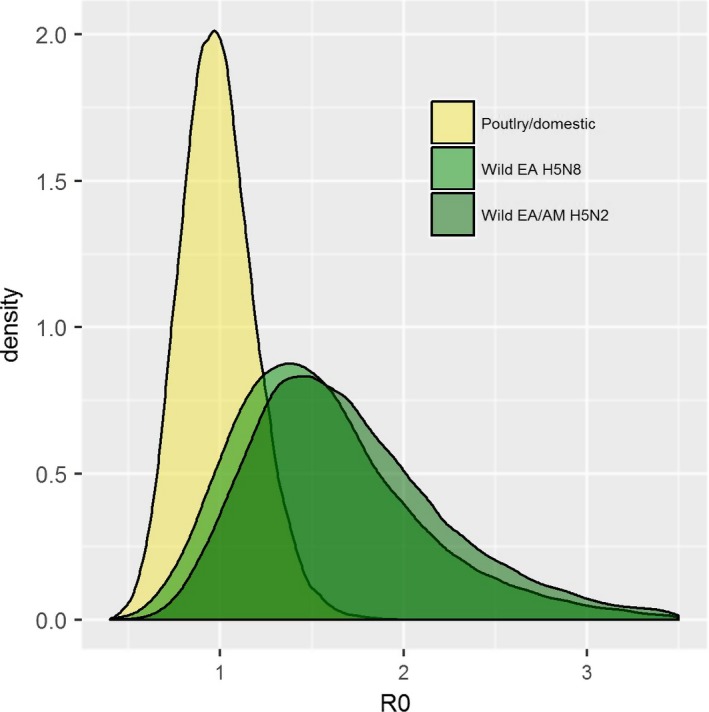
Estimates of the basic reproductive number, *R*_0_, of clade 2.3.4.4 highly pathogenic hemagglutinin (H5) segment 2014–2015 in North America. Probability density distributions represent the Bayesian posterior estimates of *R*_0_ for all poultry and domestic bird hosts sampled 2 December 2014 – 1 June 2015 and wild bird hosts by highly pathogenic avian influenza virus (HPAIV) subtype sampled 2 December 2014 – 1 February 2015 to estimate dynamics when there was epidemiological evidence of wild bird transmission (Lee et al., 2015)

The mean estimated time of the most recent common ancestor (TMRCA) for each segment was 28 October–28 November 2014, or approximately 1 month before the earliest isolate was collected (Table 1). There was >.75 probability that the common ancestor to all the isolates was from a wild bird in each segment (Table 1). We estimated that the EA/NA H5N2 HPAIVs that spread to the Midwestern USA were monophyletic and descended from the EA/NA H5N2 viruses detected in the western USA and Canada with a common ancestor node in similar position among the three segments with 0.64, 0.88, and 0.80 posterior probabilities for the common ancestor of the HA, PB2, and M segments, respectively. The estimated TMRCA of the Midwestern EA/NA H5N2 HPAIVs was 13 January 2015 to 21 February 2015 (Figures 1, S1–S2, Table 1). There was a 93% posterior probability that the Midwestern EA/NA H5N2 HPAIV HA segment common ancestor was from a domestic bird and a 54% and 53% probability for the PB2 and M segment, respectively (Table 1). We estimated that the basic reproductive number (*R*_0_) of the HPAIVs in poultry was approximately 1 and slightly higher in wild birds, with no difference between the EA H5N8 and EA/NA H5N2 subtypes (Tables 1, 2, Figure 2). In the full analysis with all sequences, as well as the nested analysis of the wild hosts, we estimated that greater than 0.75 of the viral population was sampled (mean posterior estimate, ψ) across all segments (Tables 1, 2). To estimate the *R*_0_ by host type, we also estimated the viral lineage death rate (Kuhnert et al., 2016) and estimated approximately similar rates across all segments in the full and nested analysis (Tables S2–S7).

**Table 1:** Key epidemiological and phylogenetic parameter posterior distributions (estimate [95% highest posterior density interval]) estimated^a^ from the birth–death multitype phylogenetic model of the Eurasian origin HA, PB2, and M genes of the 2014–2015 clade 2.3.4.4 highly pathogenic avian influenza outbreak in wild birds and poultry in North America from sequences isolated 2 December 2014–1 June 2015

	HA		PB2		M	
	Poultry	Wild birds	Poultry	Wild birds	Poultry	Wild birds
Median time of most recent common ancestor (MRCA)	15 Nov.(2 Nov., 25 Nov.)		11 Nov.(28 Oct., 23 Nov.)		18 Nov.(5‐Nov., 28 Nov.)	
Mean probability of MRCA identity^b^	0.25	0.75	0.13	0.87	0.21	0.79
Mean time of Midwest H5N2 MRCA^c^	9 Feb.(26 Jan., 21 Feb.)		30 Jan.(13 Jan., 14 Feb.)		5 Feb.(15 Jan., 9 Feb.)	
Mean probability of Midwest H5N2 MRCA identity^b^	0.93	0.07	0.54	0.46	0.53	0.47
Mean *R*_0_	0.94 (0.58, 1.30)	1.13 (0.76, 1.54)	0.90 (0.48, 1.34)	1.10 (0.77, 1.45)	0.97 (0.57, 1.39)	1.07 (0.74, 1.41)
Mean estimated proportion sampled	0.76 (0.42, 1.0)	0.66 (0.31, 0.99)	0.75 (0.41, 1.0)	0.60 (0.21, 1.0)	0.78 (0.44, 1.0)	0.59 (0.21, 0.99)
Median estimated number transmission events^d^						
To wild birds	10 (6, 18)		13 (8, 21)	–	9 (5, 14)	–
To poultry		7 (3, 13)	–	5 (0, 9)	–	3 (0, 7)

Tables S2–S4 contains a full set of prior distributions and posterior estimates of estimated parameters.

Probability estimated from the root node type estimated from sampled posterior phylogenetic trees. The maximum clade credibility tree for the HA gene segment is presented in Figure 1.

Estimated time of common ancestor node with HA = 0.9, PB2 = 0.85, and M = 0.51 posterior probability of the monophyletic group of H5N2 sequences isolated in the Central and Mississippi waterfowl migratory flyways (Figure 1).

Estimated number of transmission events accounts for viral migration rate between host types and relative frequencies of estimated ancestor host types in the phylogeny.

**Table 2:** Key epidemiological parameter posterior distributions (estimate and [95% highest posterior density interval]) estimated^a^ from the birth–death multitype phylodynamic model of the Eurasian origin HA, PB2, and M genes in wild birds sampled 2 December 2014–1 February 2015 from western North America of the 2014–2015 clade 2.3.4.4 highly pathogenic avian influenza outbreak in wild birds and poultry in North America

	HA		PB2		M	
	H5N8	H5N2	H5N8	H5N2	H5N8	H5N2
Mean *R*_0_	1.59 (0.67, 2.71)	1.74 (0.79, 2.93)	2.68 (0.92, 5.13)	2.95 (0.95, 5.79)	1.60 (0.56, 2.83)	1.93 (0.81, 3.36)
Mean estimated proportion sampled	0.61 (0.21, 1.0)	0.79 (0.45, 1.0)	0.53 (0.11, 0.99)	0.70 (0.28 1.0)	0.65 (0.19, 1.0)	0.75 (0.36, 1.0)

a Table S5–S7 contains a full set of prior distributions and posterior estimates of estimated parameters.

We explicitly estimated the migration rates of viral lineages from wild hosts to domestic hosts and vice versa using the full analysis and estimated that the rate from wild hosts to poultry was greater than from poultry to wild hosts (Table S2–S4). When we accounted for the frequency of ancestral states and positions of wild versus poultry isolates in the posterior sample of phylogenetic trees, we estimated that the realized number of host‐type viral migrations was 2–3× greater from poultry to wild hosts than vice versa during the outbreak (Table 1).

## 4 DISCUSSION

The foci of recent emergent highly pathogenic H5 AIVs has been in Asia where transmission among wild birds, semi‐domestic birds, and poultry is hypothesized to occur often (Chen et al., 2006; Gauthier‐Clerc, Lebarbenchon, & Thomas, 2007). One likely source of intercontinental introduction of Asian AIVs into North America is migratory waterfowl (Pearce et al., 2011), and our analysis suggests that the most recent common ancestor of the 2014–2015 HPAIVs in North America was from an infection in a wild bird (Table 1). Following the introduction, the transmission dynamics were sufficient in wild birds and poultry to maintain transmission in both systems without spillover or spillback. We estimated the basic reproduction number, *R*_0_, to be slightly higher in wild birds (mean posterior estimates 1.1–1.2) compared to *R*_0_ within poultry (mean posterior estimates ~0.90–0.97) in the joint analysis of all wild bird and poultry sequences (Figure 2). Both estimates are in line with influenza A *R*_0_ values estimated in humans using phylodynamic and traditional epidemiologic methods (Fraser et al., 2009; Kuhnert et al., 2016), wild birds (Iglesias et al., 2011), and commercial poultry where biosecurity was implemented (Garske, Clarke, & Ghani, 2007; Stegeman et al., 2004). In the analysis of only wild bird sequences during the time period when there was previously published evidence of wild bird transmission (Lee et al., 2015), *R*_0_ was similar among the EA H5N8 subtype and reassortant EA/NA H5N2 subtype in wild birds in the HA, PB2, an M segments. The mean posterior estimates of *R*_0_ for the three segments varied by subtype in wild birds; however, the 95% HPD intervals suggested a similar range of uncertainty (Table 2). The mean wild bird *R*_0_ estimates were also slightly higher for wild birds during the period in this second analysis, and we discuss the interpretation and limitations of that estimate later. Hence, our estimated phylodynamic parameters of 2014–2015 EA/NA H5Nx outbreak provided evidence against the hypotheses that (i) transmission of highly pathogenic AIVs in wild birds were restricted during this outbreak, (ii) transmission within poultry are not self‐sustaining without input from the wild bird–poultry interface, and (iii) reassortment with North American gene segments provided a selective advantage to transmission of the Eurasian gene segments in wild birds.

Our estimated basic reproductive numbers for AIV transmission in wild bird hosts explicitly incorporated phylogenetic and epidemiologic uncertainty, and it was facilitated by the intensive sampling for AIVs in wild aquatic birds in response to the detection of HPAIV (Bevins et al., 2016; Ip et al., 2016), as well as the emerging field of phylodynamics being applied to rapidly evolving pathogens (Frost & Volz, 2010; Stadler & Bonhoeffer, 2013; Stadler et al., 2012; Volz & Frost, 2014). Cryptic transmission dynamics of pathogens in wildlife are often cited as limiting to understanding the ecology and consequences of diseases at the wildlife–domestic animal and human interface (Buhnerkempe et al., 2015). We provided an application of phylodynamic methods that were able to infer key epidemiological processes from pathogen sequence data in a wildlife–poultry outbreak that are untenable using traditional wildlife and epidemiological methods. Notably, we showed that a novel HPAIV was capable of maintaining transmission among wild aquatic birds (*R*_0_ > 1) and that reassortment with AIV native to North America did not seem to alter transmission in the reservoir hosts.

An important caveat to the inference made from phylodynamic analyses of sequence data is the limitation of sampling (Baele, Suchard, Rambaut, & Lemey, 2017; Boskova, Bonhoeffer, & Stadler, 2014; Frost & Volz, 2010). Both the sampling within the spatiotemporal frame of pathogen detection and the extent of that sampling need to be carefully considered when interpreting the results of sequence analyses. A significant advance of the birth–death family of phylodynamic models is that the sampling proportion within the frame of detection is explicitly estimated (Stadler et al., 2013). By estimating the sampling effort, we can account for nonuniform sampling through time and host type (Kuhnert et al., 2016), relax the coalescent‐model assumption that our sample is small relative to the full population size (Boskova et al., 2014), and even provide information to evaluate surveillance strategies. We focused on a relatively short time period and assumed a constant sampling, but explicitly estimated sampling in wild birds versus poultry in our full analysis. We expected high rates of detection in poultry because of the high mortality rate, along with the industry and regulatory response. The high estimate of the proportion of lineage diversity sampled in wild birds (mean proportion > 0.60, Table 1) also suggested that the surveillance activities and effort were effective in response to the initial detection in terms of capturing the diversity of the outbreak genetics (Bevins et al., 2016; Ip et al., 2016).

The dynamics of the EA/NA H5Nx HPAIVs in wild birds outside of the sampling frame remains a mystery (Krauss et al., 2016). Detecting pathogen dynamics in migratory species is challenging and our detection was limited to a snapshot where intense sampling effort was fortunate to coincide with a non‐HPAIV mortality event in wild waterfowl and the last portion of the waterfowl harvest season (Ip et al., 2016). The harvest season for duck and goose harvest ended 25 January 2015 or earlier in Oregon and Washington (http://www.eregulations.com/oregon/game-bird/game-bird-seasons/; http://wdfw.wa.gov/hunting/regulations/). However, following the sampling time period migratory waterbirds were moving primarily north to south (Buhnerkempe et al., 2016) and LPAIV phylogeography in North America tends to be structured by the north–south migratory flyways (Lam et al., 2012). Factors that limit AIV transmission in wild birds, in general, include temperature dependent transmission (Brown, Goekjian, Poulson, Valeika, & Stallknecht, 2009; Farnsworth et al., 2012), population immunity (Hénaux, Parmley, Soos, & Samuel, 2013), and decrease in host density with migration to warmer climates (Hill et al., 2016; Roche et al., 2009). The hypothesis that the apparent disappearance of the 2014–2015 HPAIVs in wild birds stemmed from unobserved mechanisms that suppressed transmission capacity of highly pathogenic phenotypes in wild bird hosts does not appear consistent with the data in our sampling frame (Krauss et al., 2016). Hence, we suggest an alternative hypothesis that novel trans‐continental AIV genotypes appear rarely because of stochastic effects of limiting transmission factors acting on small viral population sizes, not specific to HPAIVs.

The interpretation of transmission dynamics from the poultry sequences was slightly less clear than for wild birds. Our data were a single sequence each from a subset of all the infected poultry facilities, and our interpretation is that the transmission dynamics represented facility‐to‐facility transmission. The EA/NA H5N2 outbreak was primarily in high‐density turkey and egg production facilities, and transmission was likely explosive within a facility. We did not explicitly account for any of the evolutionary dynamics at the within‐facility scale, although the consequences were borne out through observations of rapid mortality (USDA APHIS, 2015). Hence, our estimate that *R*_0_ was indistinguishable from one among the infected poultry suggests that facility‐to‐facility transmission was just enough to keep the outbreak alive but not enough to create a truly explosive epizootic. Our analysis could not evaluate mechanisms of spread among the Midwest US poultry facilities, including the potential role of wild birds. However, we estimated that the poultry sequences sampled a high proportion of viral diversity in all segments (Table 1). We cannot rule out that an unobserved reservoir of HPAIVs existed outside the sample associated with Midwest, but other surveillance in wild birds on and near infected poultry facilities detected no HPAIVs in wild birds contemporary to the poultry outbreak (Jennelle et al., 2016) and limited exposure in wildlife (Grear, Dusek, Walsh, & Hall, 2017; Shriner et al., 2016). A further observation from the phylogeny of the Midwestern poultry sequences suggested that transmission was not structured by production type, with egg‐laying chicken and domestic turkey facilities represented within the same viral lineages (Figures 1, S1–S2).

Our phylodynamic analysis and similar analyses from others indicated that transmission likely occurred among and between wild birds and poultry at some time before or early during the time period when these HPAIVs were first detected (Figure 1; Lee et al., 2015). However, the frequency and direction of individual events remain uncertain using either traditional methods or this phylodynamic approach to the epidemiology of the outbreak (USDA APHIS, 2015). We were able to make some inference to cross‐species transmission at a coarse scale: notably, the likely host type of the common ancestor to all sequenced isolates was likely a wild bird, and the host type of the well‐supported nodes that defined the clade of EA/NA H5N2 that affected Midwest poultry facilities was a domestic bird (Figure 1). The infected wild hosts in the Midwestern EA/NA H5N2 clade were raptors and geese and all were found with evidence of HPAI disease‐related mortality, suggesting that these individuals were not involved in onward transmission (Ip et al., 2015, 2016).

### 4.1 Interpretation of transmission parameters

The epidemiological parameters estimated form the birth–death family of phylodynamic models share a basis with familiar host‐transition compartmental models of disease dynamics that focus on the host population (Kermack & McKendrick, 1927). However, there are key differences in the interpretation of parameters because phylodynamic models focus on the population dynamics of the pathogen (HPAI viral segments in our case) and may not include or require much host information. Hence, the most relevant parameter is *R*_0_ because it is a unitless parameter for epidemic growth. Whether the derivation for *R*_0_ is based on host dynamics (transmission rate and infectious period) or viral dynamics (“births and deaths” of viral lineages), *R*_0_ infers whether infected hosts or viral lineages (the population unit of interest) are replacing themselves fast enough for an epidemic to persist and grow (Mccallum, Barlow, & Hone, 2001; Stadler et al., 2012). The comparison of *R*_0_ based on different analytical methods and data is best interpreted relative to the threshold of *R*_0_ = 1; where *R*_0_ > 1 infers an outbreak is growing and *R*_0_ < 1 infers an outbreak is dying out. Comparisons of *R*_0_ magnitude across data types and methods should be considered carefully because the definition of what is growing (i.e., population of viral lineages or population of infected hosts) may have different epidemiological consequences. Nonetheless, our inference that the EA/NA H5N2 (HA segment mean *R*_0_ = 1.7, 95% HPD [0.8, 2.9]) and EA H5N8 (HA segment mean *R*_0_ = 1.6, 95% HPD [0.7, 2.7]) was in an outbreak state in wild bird reservoir hosts and persisting around the threshold for an outbreak (HA segment mean *R*_0_ = 0.94, 95% HPD [0.76, 1.54]) in poultry, still provide valuable insights where traditional host‐based data on infection dynamics were lacking.

### 4.2 Conclusions and applications

The increasing availability of genomic data provides a valuable tool to integrate with traditional epidemiology. We did not find evidence to support the hypothesis that transmission of the clade 2.3.4.4 HPAIVs was restricted in wild reservoir hosts. Our estimates of the effective reproductive number in wild hosts suggested that transmission was ongoing and above the threshold to persist (mean *R*_0_ > 1, Figure 2, Tables S2–S7) and we did not find evidence to support the hypothesis that reassortment for the presumptive Eurasian source H5N8 with North American LPAIVs provided any evolutionary advantage to reassortant lineages in wild birds. We also did not find evidence that input from wild birds played a role in the outbreak of the EA/NA H5N2 outbreak in Midwestern poultry. Instead, our analysis suggested that once the EA/NA H5N2 lineage entered the poultry production system in the Midwest USA, transmission was driven through poultry production‐related mechanisms because we found close phylogenetic distance among sequences from poultry facilities (Figure 1), relatively infrequent estimate of cross‐species transmission (Table 1), high estimated proportion of viral diversity that was sampled (Table 1), and other surveillance data failed to detect this lineage in reservoir hosts (Grear et al., 2017; Ip et al., 2016; Jennelle et al., 2016; Krauss et al., 2016). We suggest that the lack of detection in wild birds points to facility biosecurity that was nearly sufficient to reduce the epidemic size, but had just enough failures to produce the observed consequences (50M birds depopulated and over US $3 billion; Greene, 2015). Thus, examining finer scale epidemiologic patterns of poultry facility infection will likely be fruitful if results can direct biosecurity improvements that reduce the transmission rate and increase detection rate. Lapses in biosecurity that allow virus to spread with people or equipment and airborne dissemination of viral particles from infected farms are two hypotheses for poultry spread (USDA APHIS, 2015). The selective pressures placed on viruses that spread between farms are likely very different from within‐farm transmission, especially within and between high‐density poultry production facilities. In addition to informing the mechanism of spread, more intensive sampling in poultry has the potential to detect the outcome of different selective pressures that could expand or restrict viral diversity and have consequences for vaccine application and adaptation to human infection.

Phylodynamic tools can provide insights into joint evolutionary‐epidemiological processes. The emergence of genetic sequencing as a standard tool in epidemiologic investigations is providing the raw data to use phylodynamics in an epidemiological toolbox; especially to investigate wildlife diseases. However, the most efficient use of these cutting‐edge tools requires additional work to incorporate evolutionary principles with traditional epidemiology to form and test hypotheses that can be translated into disease control actions. Type A avian influenza ecology and evolution represent a relatively data rich topic at the interface of wildlife, domestic animal, and human disease. Future work could focus on using such cutting‐edge phylodynamic methods to test hypotheses about geographic spread of AIVs in wild birds, multiyear evolutionary processes of AIVs in reservoir hosts, and relative fitness of highly pathogenic versus low‐pathogenic AIVs in wild birds.

## DATA ARCHIVING

Sequence data for this study are available at Influenza Research Database (IRD): http://www.fludb.org. Summary phylogenetic tree files and.xml file with the phylodynamic model setup are available on Dryad.org, https://doi.org/10.5061/dryad.247st.
