# Supplementary material for: Inferring epidemiologic dynamics from viral evolution: 2014–2015 Eurasian/North American highly pathogenic avian influenza viruses exceed transmission threshold, R 0 = 1, in wild birds and poultry in North America
Source: Evol Appl. 2017 Dec 1;11(4):547–57. doi: 10.1111/eva.12576 (PMC5891053; doi:10.1111/eva.12576)

Table S1- sequence data

| StrainName | HA Sequence Accession | PB2 Sequence Accession | M Sequence Accession | Subtype | Collection Date | State or Province | Species | host type full analysis | host type wild bird analysis |
| --- | --- | --- | --- | --- | --- | --- | --- | --- | --- |
| A/turkey/BC/FAV10/2014 | KP307957 | KP307954 | KP307960 | H5N2 | 2-Dec-2014 | British Columbia | Turkey | poultry | NA |
| A/chicken/BC/FAV8/2014 | KP795729 | KP307973 | KP307979 | H5N2 | 3-Dec-2014 | British Columbia | Chicken | poultry | NA |
| A/chicken/BC/FAV9/2014 | KP795737 | KP307981 | KP307987 | H5N2 | 3-Dec-2014 | British Columbia | Chicken | poultry | NA |
| A/Northern pintail/Washington/40964/2014 | KP307976 | KP739378 | KP739384 | H5N2 | 8-Dec-2014 | Washington | Northern Pintail | wild | H5N2 |
| A/gyrfalcon/Washington/41088-6/2014 | KP307984 | KP739386 | KP739392 | H5N8 | 8-Dec-2014 | Washington | Falcon | wild | H5N8 |
| A/mallard/Washington/195246/2014 | KU201896 | KP739394 | KP739400 | H5N2 | 8-Dec-2014 | Washington | Mallard | wild | H5N2 |
| A/chicken/Oregon/41613-2/2014 | KP739405 | KP739402 | KP739408 | H5N8 | 16-Dec-2014 | Oregon | Chicken | poultry | NA |
| A/guinea fowl/Oregon/41613-1/2014 | KP739413 | KP739410 | KP739416 | H5N8 | 16-Dec-2014 | Oregon | Guineafowl | poultry | NA |
| A/American wigeon/Washington/195198/2014 | KU201880 | KP739418 | KP739424 | H5N8 | 16-Dec-2014 | Washington | American Wigeon | wild | H5N8 |
| A/American wigeon/Washington/195205/2014 | KU201888 | KP795726 | KP795732 | H5N8 | 16-Dec-2014 | Washington | American Wigeon | wild | H5N8 |
| A/mallard/Oregon/AH0003821/2014 | KU201784 | KP795734 | KP795740 | H5N2 | 20-Dec-2014 | Oregon | Mallard | wild | H5N2 |
| A/mallard/Idaho/AH0005954/2014 | KU201768 | KP892988 | KP892994 | H5N8 | 22-Dec-2014 | Idaho | Mallard | wild | H5N8 |
| A/mallard/Idaho/AH0005955/2014 | KU201776 | KR150898 | KR150904 | H5N8 | 22-Dec-2014 | Idaho | Mallard | wild | H5N8 |
| A/mallard/Oregon/195547/2014 | KU201816 | KR150906 | KR150912 | H5N2 | 22-Dec-2014 | Oregon | Mallard | wild | H5N2 |
| A/Northern pintail/Washington/195365/2014 | KU201808 | KR233979 | KR233985 | H5N2 | 23-Dec-2014 | Washington | Northern Pintail | wild | H5N2 |
| A/mallard/Washington/195810/2014 | KU201912 | KR233987 | KR233993 | H5N2 | 23-Dec-2014 | Washington | Mallard | wild | H5N2 |
| A/mallard/Oregon/195536/2014 | KU201872 | KR233995 | KR234001 | H5N8 | 24-Dec-2014 | Oregon | Mallard | wild | H5N8 |
| A/American green-winged teal/Washington/195750/2014 | KP739421 | KR234003 | KR234009 | H5N1 | 29-Dec-2014 | Washington | Green-Winged Teal | wild | NA |
| A/Northern pintail/Washington/196271/2014 | KU201840 | KR234011 | KR234017 | H5N8 | 29-Dec-2014 | Washington | Northern Pintail | wild | H5N8 |
| A/peregrine falcon/Washington/196426/2014 | KU201904 | KR234019 | KR234025 | H5N8 | 29-Dec-2014 | Washington | Peregrine Falcon | wild | H5N8 |
| A/chicken/Washington/61-9/2014 | KP739381 | KR234027 | KR234033 | H5N2 | 30-Dec-2014 | Washington | Chicken | poultry | NA |
| A/domestic duck/Washington/61-16/2014 | KP739389 | KR234035 | KR234041 | H5N2 | 30-Dec-2014 | Washington | Domestic Duck | poultry | NA |
| A/turkey/Washington/61-22/2014 | KP739397 | KR492971 | KR492977 | H5N2 | 30-Dec-2014 | Washington | Turkey | poultry | NA |
| A/American wigeon/Washington/195968/2014 | KU201824 | KT002470 | KT002476 | H5N8 | 30-Dec-2014 | Washington | American Wigeon | wild | H5N8 |
| A/Canada goose/Washington/197619/2014 | KU201920 | KT002478 | KT002484 | H5N2 | 30-Dec-2014 | Washington | Canada Goose | wild | H5N2 |
| A/mallard/Washington/196262/2015 | KU201832 | KT002486 | KT002492 | H5N2 | 1-Jan-2015 | Washington | Mallard | wild | H5N2 |
| A/American wigeon/Utah/AH0007824/2015 | KU201616 | KT002494 | KT002500 | H5N8 | 2-Jan-2015 | Utah | American Wigeon | wild | H5N8 |
| A/American wigeon/Washington/196336/2015 | KU201856 | KT002502 | KT002508 | H5N1 | 2-Jan-2015 | Washington | American Wigeon | wild | NA |
| A/American wigeon/Washington/196340/2015 | KU201864 | KT002510 | KT002516 | H5N1 | 2-Jan-2015 | Washington | American Wigeon | wild | NA |
| A/wood duck/Oregon/AH0007244/2015 | KU201640 | KT002518 | KT002524 | H5N2 | 3-Jan-2015 | Oregon | Wood Duck | wild | H5N2 |
| A/wood duck/Oregon/AH0007257/2015 | KU201648 | KT002526 | KT002531 | H5N2 | 3-Jan-2015 | Oregon | Wood Duck | wild | H5N2 |
| A/wood duck/Oregon/AH0007263/2015 | KU201656 | KT280929 | KT280935 | H5N2 | 3-Jan-2015 | Oregon | Wood Duck | wild | H5N2 |
| A/Northern shoveler/Oregon/AH0007332/2015 | KU201664 | KT762881 | KT762887 | H5N2 | 3-Jan-2015 | Oregon | Northern Shoveler | wild | H5N2 |
| A/Northern shoveler/Oregon/AH0007337/2015 | KU201672 | KT762889 | KT762895 | H5N2 | 3-Jan-2015 | Oregon | Northern Shoveler | wild | H5N2 |
| A/Northern shoveler/Oregon/AH0007339/2015 | KU201680 | KT762897 | KT762903 | H5N2 | 3-Jan-2015 | Oregon | Northern Shoveler | wild | H5N2 |
| A/mallard/Oregon/AH0003952/2015 | KU201624 | KT762905 | KT762911 | H5N2 | 5-Jan-2015 | Oregon | Mallard | wild | H5N2 |
| A/Northern pintail/Oregon/AH0003967/2015 | KU201632 | KT762913 | KT762919 | H5N2 | 5-Jan-2015 | Oregon | Mallard | wild | H5N2 |
| A/mallard/Idaho/AH0008597/2015 | KU201688 | KT762921 | KT762927 | H5N8 | 7-Jan-2015 | Idaho | Mallard | wild | H5N8 |
| A/mallard/Oregon/AH0008887/2015 | KU201696 | KT762929 | KT762935 | H5N2 | 7-Jan-2015 | Oregon | Mallard | wild | H5N2 |
| A/mallard/Washington/196865/2015 | KU201848 | KT762937 | KT762943 | H5N2 | 10-Jan-2015 | Washington | Mallard | wild | H5N2 |
| A/Northern pintail/Oregon/AH0003871/2015 | KU201704 | KT762945 | KT762951 | H5N2 | 12-Jan-2015 | Oregon | Northern Pintail | wild | H5N2 |
| A/American green-winged teal/Oregon/AH0012403/2015 | KU201736 | KT762953 | KT762959 | H5N2 | 14-Jan-2015 | Oregon | Green Winged Teal | wild | H5N2 |
| A/mallard/Idaho/AH0007412/2015 | KU201712 | KU201605 | KU201611 | H5N2 | 17-Jan-2015 | Idaho | Mallard | wild | H5N2 |
| A/mallard/Idaho/AH0007413/2015 | KU201720 | KU201613 | KU201619 | H5N2 | 17-Jan-2015 | Idaho | Mallard | wild | H5N2 |
| A/American green-winged teal/Idaho/AH0011899/2015 | KU201728 | KU201621 | KU201627 | H5N2 | 18-Jan-2015 | Idaho | Green Winged Teal | wild | H5N2 |
| A/Canada goose/Oregon/AH0012452/2015 | KU201744 | KU201629 | KU201635 | H5N8 | 18-Jan-2015 | Oregon | Canada Goose | wild | H5N8 |
| A/American wigeon/Oregon/AH0012525/2015 | KU201752 | KU201637 | KU201643 | H5N8 | 18-Jan-2015 | Oregon | American Wigeon | wild | H5N8 |
| A/bald eagle/Idaho/15-002892-2/2015 | KU201800 | KU201645 | KU201651 | H5N8 | 20-Jan-2015 | Idaho | Bald Eagle | wild | H5N8 |
| A/turkey/California/K1500169-1.2/2015 | KR150901 | KU201653 | KU201659 | H5N8 | 21-Jan-2015 | California | Turkey | poultry | NA |
| A/mallard/Nevada/AH0006855/2015 | KU201760 | KU201661 | KU201667 | H5N8 | 23-Jan-2015 | Nevada | Mallard | wild | H5N8 |
| A/pheasant/Washington/3147-2/2015 | KR150909 | KU201669 | KU201675 | H5N2 | 26-Jan-2015 | Washington | Pheasant | poultry | NA |
| A/chicken/Washington/3490-18/2015 | KR233990 | KU201677 | KU201683 | H5N2 | 29-Jan-2015 | Washington | Chicken | poultry | NA |
| A/chicken/BC/FAV2/2015 | KP892991 | KU201685 | KU201691 | H5N1 | 6-Feb-2015 | British Columbia | Chicken | poultry | NA |
| A/chicken/California/15-004912/2015 | KU201792 | KU201693 | KU201699 | H5N8 | 6-Feb-2015 | California | Chicken | poultry | NA |
| A/chicken/Oregon/A01819044/2015 | KR233998 | KU201701 | KU201707 | H5N2 | 11-Feb-2015 | Oregon | Chicken | poultry | NA |
| A/turkey/Minnesota/7172-1/2015 | KR234006 | KU201709 | KU201715 | H5N2 | 27-Feb-2015 | Minnesota | Turkey | poultry | NA |
| A/turkey/Missouri/7458-1/2015 | KR234014 | KU201717 | KU201723 | H5N2 | 6-Mar-2015 | Missouri | Turkey | poultry | NA |
| A/turkey/Arkansas/7791-1/2015 | KR234022 | KU201725 | KU201731 | H5N2 | 8-Mar-2015 | Arkansas | Turkey | poultry | NA |
| A/chicken/Kansas/8395-3/2015 | KR234030 | KU201733 | KU201739 | H5N2 | 12-Mar-2015 | Kansas | Chicken | poultry | NA |
| A/Canada goose/Kansas/197850/2015 | KU201943 | KU201741 | KU201747 | H5N2 | 13-Mar-2015 | Kansas | Canada Goose | wild | NA |
| A/snow goose/Missouri/15-011246-1/2015 | KU201608 | KU201925 | KU201931 | H5N2 | 15-Mar-2015 | Missouri | Snow Goose | wild | NA |
| A/turkey/Minnesota/9845-4/2015 | KR234038 | KU201749 | KU201755 | H5N2 | 25-Mar-2015 | Minnesota | Turkey | poultry | NA |
| A/turkey/Minnesota/9892-2/2015 | KR233982 | KU201757 | KU201763 | H5N2 | 26-Mar-2015 | Minnesota | Turkey | poultry | NA |
| A/turkey/South Dakota/15-010371/2015 | KT762932 | KU201765 | KU201771 | H5N2 | 30-Mar-2015 | South Dakota | Turkey | poultry | NA |
| A/chicken/Montana/15-010559-1/2015 | KT762884 | KU201773 | KU201779 | H5N2 | 1-Apr-2015 | Montana | Chicken | poultry | NA |
| A/chicken/Wisconsin/15-011595-1/2015 | KT762948 | KU201781 | KU201787 | H5N2 | 8-Apr-2015 | Wisconsin | Chicken | poultry | NA |
| A/turkey/North Dakota/15-011420-13/2015 | KT762892 | KU201789 | KU201795 | H5N2 | 10-Apr-2015 | North Dakota | Turkey | poultry | NA |
| A/turkey/Iowa/11762-1/2015 | KT002473 | KU201797 | KU201803 | H5N2 | 12-Apr-2015 | Iowa | Turkey | poultry | NA |
| A/chicken/Wisconsin/15-012160-1/2015 | KT762956 | KU201805 | KU201811 | H5N2 | 13-Apr-2015 | Wisconsin | Chicken | poultry | NA |
| A/snowy owl/Wisconsin/198399/2015 | KU201936 | KU201813 | KU201819 | H5N2 | 13-Apr-2015 | Wisconsin | Snowy Owl | wild | NA |
| A/turkey/Wisconsin/15-012012-2/2015 | KT762908 | KU201821 | KU201827 | H5N2 | 14-Apr-2015 | Wisconsin | Turkey | poultry | NA |
| A/Cooper's hawk/Minnesota/198225/2015 | KU201928 | KU201829 | KU201835 | H5N2 | 14-Apr-2015 | Minnesota | Cooper's Hawk | wild | NA |
| A/chicken/Iowa/04-20/2015 | KR492974 | KU201837 | KU201843 | H5N2 | 20-Apr-2015 | Iowa | Chicken | poultry | NA |
| A/turkey/North Dakota/15-013049-1/2015 | KT762916 | KU201845 | KU201851 | H5N2 | 21-Apr-2015 | North Dakota | Turkey | poultry | NA |
| A/turkey/Iowa/13541-1/2015 | KT002481 | KU201853 | KU201859 | H5N2 | 25-Apr-2015 | Iowa | Turkey | poultry | NA |
| A/chicken/Iowa/13542-2/2015 | KT002489 | KU201861 | KU201867 | H5N2 | 25-Apr-2015 | Iowa | Chicken | poultry | NA |
| A/chicken/Minnesota/15-013533-1/2015 | KT762924 | KU201869 | KU201875 | H5N2 | 26-Apr-2015 | Minnesota | Chicken | poultry | NA |
| A/chicken/Iowa/21981/2015 | KT280932 | KU201877 | KU201883 | H5N2 | 27-Apr-2015 | Iowa | Chicken | poultry | NA |
| A/turkey/Iowa/14318-1/2015 | KT002497 | KU201885 | KU201891 | H5N2 | 30-Apr-2015 | Iowa | Turkey | poultry | NA |
| A/chicken/Iowa/14399-4/2015 | KT002521 | KU201893 | KU201899 | H5N2 | 30-Apr-2015 | Iowa | Chicken | poultry | NA |
| A/turkey/Iowa/14319-1/2015 | KT002505 | KU201901 | KU201907 | H5N2 | 1-May-2015 | Iowa | Turkey | poultry | NA |
| A/chicken/Iowa/14322-6/2015 | KT002513 | KU201909 | KU201915 | H5N2 | 1-May-2015 | Iowa | Chicken | poultry | NA |
| A/chicken/Iowa/14589-1/2015 | KT002528 | KU201917 | KU201923 | H5N2 | 4-May-2015 | Iowa | Chicken | poultry | NA |
| A/chicken/Nebraska/15-017990-5/2015 | KT762940 | KU201933 | KU201939 | H5N2 | 29-May-2015 | Nebraska | Chicken | poultry | NA |
| A/chicken/Nebraska/15-017897-1/2015 | KT762900 | KU201941 | KU201946 | H5N2 | 1-Jun-2015 | Nebraska | Chicken | poultry | NA |

TableS2 – Prior distributions and posterior estimates of phylogenetic and epidemic parameters for the hemagglutinin (HA) nucleotide sequences isolated from wild birds and poultry (Fig. 1) during the highly pathogenic avian influenza virus outbreak on North America, November 2014 – June 2015. Posterior estimates obtained from combining 4 independent Markov-chain Monte Carlo runs of 8-10 million iterations after discarding 10% burn-in and sampling every 1,000^th^ iteration.

| Parameter^a^ | Prior Distribution | Posterior Estimate mean [95% HPD] | Effective sample size^†^ | Gelman-Rubin potential scale reduction factor 95% upper bound |
| --- | --- | --- | --- | --- |
| **Epidemiological** |  |  |  |  |
| R_0_ wild | lnNorm(0, 1.5) | 1.13 [0.76, 1.54] | 4659 | 1.00 |
| R_0_ poultry | lnNorm(0, 1.5) | 0.94 [0.58, 1.30] | 2988 | 1.00 |
| Lineage death rate (year^-1^) | lnNorm(2.5, 1) | 27.1 [17.9, 37.8] | 1386 | 1.00 |
| Wild to poultry virus migration rate ( year^-1^) | Exp (1) | 6.33 [2.20, 11.3] | 1513 | 1.00 |
| Poultry to wild virus migration rate (year^-1^) | Exp (1) | 3.67 [0.72, 7.37] | 1372 | 1.00 |
| Sampling proportion wild | Beta(1, 1) | 0.66 [0.31, 0.99] | 3029 | 1.00 |
| Sampling proportion poultry | Beta(1, 1) | 0.76 [0.42, 1.0] | 2283 | 1.00 |
| **Phylogenetic** |  |  |  |  |
| Proportion invariant nucleotide sites | Unif(0, 1) | 0.20 [4.2E-4, 0.44] | 1216 | 1.00 |
| uncorrelated relaxed lognormal molecular clock rate mean | lnNorm(-6.2, 3.5) | 0.021 [0.014, 0.030] | 871 | 1.00 |
| uncorrelated relaxed lognormal molecular clock rate standard deviation | Gamma(0.540, 0.385) | 0.80 [0.40, 1.21] | 1359 | 1.00 |
| A to C nucleotide transition rate | Gamma(0.05, 10) | 0.14 [0.06. 0.25] | 2343 | 1.00 |
| A to G nucleotide transition rate | Gamma(0.05, 10) | 0.60 [0.38, 0.88] | 2184 | 1.00 |
| A to T nucleotide transition rate | Gamma(0.05, 10) | 0.04 [6.9E-3, 0.09] | 1687 | 1.01 |
| C to G nucleotide transition rate | Gamma(0.05, 10) | 0.04 [3.6E-4, 0.11] | 1423 | 1.00 |
| G to T nucleotide transition rate | Gamma(0.05, 10) | 0.09 [0.02, 0.16] | 1481 | 1.00 |
| C to T nucleotide transition rate | 1^‡^ |  |  |  |
| * Parameters used to specified in birth-death multitype model template implemented in program Beauti v2.4.3 (Bouckaert et al., 2014; Kuhnert et al., 2016) | | | | |
| ^†^ Effective sample size calculated in program Tracer v.1.6.0 (Rambaut, Suchard, Xie, & Drummond, 2014) | | | | |
| ^‡^ C to T nucleotide transition rate held constant and other nucleotide transition rates estimated relative to it in the General Time Reversible (GTR) +Γ_4_ substitution model | | | | |

TableS3 – Prior distributions and posterior estimates of phylogenetic and epidemic parameters for the polymerase subunit PB2 nucleotide sequences isolated from wild birds and poultry (Fig. S1) during the highly pathogenic avian influenza virus outbreak on North America, November 2014 – June 2015. Posterior estimates obtained from combining 4 independent Markov-chain Monte Carlo runs of 8-10 million iterations after discarding 10% burn-in and sampling every 1,000^th^ iteration.

| Parameter* | Prior Distribution | Posterior Estimate mean [95% HPD] | Effective sample size† | Gelman-Rubin potential scale reduction factor 95% upper bound |
| --- | --- | --- | --- | --- |
| **Epidemiological** |  |  |  |  |
| R_0_ wild | lnNorm(0, 1.5) | 1.10 [0.77, 1.45] | 7345 | 1.00 |
| R_0_ poultry | lnNorm(0, 1.5) | 0.90 [0.48, 1.34] | 380 | 1.01 |
| Lineage death rate (year^-1^) | lnNorm(2.5, 1) | 23.5 [14.2, 35.0] | 520 | 1.00 |
| Wild to poultry virus migration rate ( year^-1^) | Exp (1) | 1.93 [1.65E-4, 4.04] | 391 | 1.01 |
| Poultry to Wild virus migration rate (year^-1^) | Exp (1) | 3.56 [1.51, 6.01] | 3336 | 1.00 |
| Sampling proportion wild | Beta(1, 1) | 0.60 [0.21, 1.0] | 268 | 1.00 |
| Sampling proportion poultry | Beta(1, 1) | 0.75 [0.41, 1.0] | 2051 | 1.02 |
| **Phylogenetic** |  |  |  |  |
| Proportion invariant nucleotide sites | Unif(0, 1) | 0.45 [0.08, 0.73] | 757 | 1.02 |
| uncorrelated relaxed lognormal molecular clock rate mean | lnNorm(-5.75, 3.5) | 0.010 [7.32E-3, 0.013] | 512 | 1.01 |
| uncorrelated relaxed lognormal molecular clock rate standard deviation | Gamma(0.540, 0.385) | 0.15 [4.20E-10, 0.47] | 2594 | 1.00 |
| A to C nucleotide transition rate | Gamma(0.05, 10) | 0.95 [0.02, 0.20] | 1537 | 1.00 |
| A to G nucleotide transition rate | Gamma(0.05, 10) | 1.14 [0.66, 1.74] | 1217 | 1.01 |
| A to T nucleotide transition rate | Gamma(0.05, 10) | 0.06, [9.13E-3, 0.15] | 1405 | 1.00 |
| C to G nucleotide transition rate | Gamma(0.05, 10) | 0.03 [5.40E-6, 0.10] | 759 | 1.04 |
| G to T nucleotide transition rate | Gamma(0.05, 10) | 0.14 [0.03, 0.28] | 1518 | 1.01 |
| C to T nucleotide transition rate | 1^‡^ |  |  |  |
| * Parameters used to specified in birth-death multitype model template implemented in program Beauti v2.4.3 (Bouckaert et al., 2014; Kuhnert et al., 2016) | | | | |
| ^†^ Effective sample size calculated in program Tracer v.1.6.0 (Rambaut et al., 2014) | | | | |
| ^‡^ C to T nucleotide transition rate held constant and other nucleotide transition rates estimated relative to it in the General Time Reversible (GTR) +Γ_4_ substitution model | | | | |

TableS4 – Prior distributions and posterior estimates of phylogenetic and epidemic parameters for the matrix protein (M) nucleotide sequences isolated from wild birds and poultry (Fig. S2) during the highly pathogenic avian influenza virus outbreak on North America, November 2014 – June 2015. Posterior estimates obtained from combining 4 independent Markov-chain Monte Carlo runs of 10 million iterations after discarding 10% burn-in and sampling every 1,000^th^ iteration.

| Parameter* | Prior Distribution | Posterior Estimate mean [95% HPD] | Effective sample size^†^ | Gelman-Rubin potential scale reduction factor 95% upper bound |
| --- | --- | --- | --- | --- |
| **Epidemiological** |  |  |  |  |
| R_0_ wild | lnNorm(0, 1.5) | 1.07 [0.74, 1.41] | 793 | 1.00 |
| R_0_ poultry | lnNorm(0, 1.5) | 0.97 [0.57, 1.39] | 1851 | 1.01 |
| Lineage death rate (year^-1^) | lnNorm(2.5, 1) | 33.5 [20.2, 49.0] | 597 | 1.01 |
| Wild to poultry virus migration rate ( year^-1^) | Exp (1) | 3.29 [1.08, 5.88] | 1610 | 1.00 |
| Poultry to Wild virus migration rate (year^-1^) | Exp (1) | 1.89 [2.10E-3, 4.34] | 814 | 1.00 |
| Sampling proportion wild | Beta(1, 1) | 0.59 [0.21, 0.99] | 610 | 1.00 |
| Sampling proportion poultry | Beta(1, 1) | 0.78 [0.44, 1.0] | 4354 | 1.00 |
| **Phylogenetic** |  |  |  |  |
| Proportion invariant nucleotide sites | Unif(0, 1) | 0.59 [0.13, 0.96] | 697 | 1.01 |
| uncorrelated relaxed lognormal molecular clock rate mean | lnNorm(-6.1, 3.5) | 9.57E-3 [5.54E-3, 0.014] | 793 | 1.01 |
| uncorrelated relaxed lognormal molecular clock rate standard deviation | Gamma(0.540, 0.385) | 0.17 [1.83E-8. 0.59] | 4385 | 1.00 |
| A to C nucleotide transition rate | Gamma(0.05, 10) | 0.14 [2.52E-3, 0.36] | 1175 | 1.01 |
| A to G nucleotide transition rate | Gamma(0.05, 10) | 0.68 [0.21, 1.28] | 1356 | 1.01 |
| A to T nucleotide transition rate | Gamma(0.05, 10) | 0.06 [3.49E-6, 0.20] | 792 | 1.02 |
| C to G nucleotide transition rate | Gamma(0.05, 10) | 4.49E-3 [2.0E-19, 0.02] | 129 | 1.04 |
| G to T nucleotide transition rate | Gamma(0.05, 10) | 0.15 [4.09E-3, 0.37] | 1023 | 1.01 |
| C to T nucleotide transition rate | 1^‡^ |  |  |  |
| * Parameters used to specified in birth-death multitype model template implemented in program Beauti v2.4.3 (Bouckaert et al., 2014; Kuhnert et al., 2016) | | | | |
| ^†^ Effective sample size calculated in program Tracer v.1.6.0 (Rambaut et al., 2014) | | | | |
| ^‡^ C to T nucleotide transition rate held constant and other nucleotide transition rates estimated relative to it in the General Time Reversible (GTR) +Γ_4_ substitution model | | | | |

Table S5 – Prior distributions and posterior estimates of phylogenetic and epidemic parameters for the hemagglutinin (HA) nucleotide sequences isolated from wild birds and typed based on Eurasian source H5N8 and Eurasian/North American reassortant H5N2 isolated prior to 1 February 2015 during the highly pathogenic avian influenza virus outbreak on North America, November 2014 – June 2015. Posterior estimates obtained from combining 4 independent Markov-chain Monte Carlo runs of 10 million iterations after discarding 10% burn-in and sampling every 1,000^th^ iteration.

| Parameter* | Prior Distribution | Posterior Estimate mean [95% HPD] | Effective sample size^†^ | Gelman-Rubin potential scale reduction factor 95% upper bound |
| --- | --- | --- | --- | --- |
| **Epidemiological** |  |  |  |  |
| R_0_ H5N2 | lnNorm(0, 1.5) | 1.74  [0.79, 2.93] | 6,432 | 1.00 |
| R_0_ H5N8 | lnNorm(0, 1.5) | 1.59  [0.67, 2.71] | 6,907 | 1.00 |
| Lineage death rate (year^-1^) | lnNorm(2.5, 1) | 18.3  [6.93, 31.2] | 2,429 | 1.00 |
| H5N2 to H5N8 virus migration rate ( year^-1^) | Exp (1) | 0.75  [3.5E-5, 2.21] | 3,781 | 1.00 |
| H5N8 to H5N2 virus migration rate (year^-1^) | Exp (1) | 0.88  [4.64E-4, 2.30] | 4,269 | 1.00 |
| Sampling proportion H5N2 | Beta(1, 1) | 0.79  [0.45, 1.0] | 7,914 | 1.00 |
| Sampling proportion H5N8 | Beta(1, 1) | 0.61  [0.21, 1.0] | 3,970 | 1.00 |
| **Phylogenetic** |  |  |  |  |
| Proportion invariant nucleotide sites | Unif(0, 1) | 0.29  [2.45E-4, 0.62] | 955 | 1.01 |
| uncorrelated relaxed lognormal molecular clock rate mean | lnNorm(-6.2, 3.5) | 0.02  [0.01, 0.03] | 1,882 | 1.01 |
| uncorrelated relaxed lognormal molecular clock rate standard deviation | Gamma(0.540, 0.385) | 0.53  [1.06E-7, 1.08] | 2,617 | 1.00 |
| A to C nucleotide transition rate | Gamma(0.05, 10) | 0.14  [0.03, 0.28] | 1,563 | 1.00 |
| A to G nucleotide transition rate | Gamma(0.05, 10) | 0.54  [0.28, 0.88] | 1,886 | 1.00 |
| A to T nucleotide transition rate | Gamma(0.05, 10) | 0.04  [7.55E-4, 0.11] | 1,220 | 1.00 |
| C to G nucleotide transition rate | Gamma(0.05, 10) | 0.09  [7.76E-4, 0.21] | 1,400 | 1.00 |
| G to T nucleotide transition rate | Gamma(0.05, 10) | 0.16  [0.04, 0.32] | 1,656 | 1.00 |
| C to T nucleotide transition rate | 1^‡^ |  |  |  |
| * Parameters used to specified in birth-death multitype model template implemented in program Beauti v2.4.3 (Bouckaert et al., 2014; Kuhnert et al., 2016) | | | | |
| ^†^ Effective sample size calculated in program Tracer v.1.6.0 (Rambaut et al., 2014) | | | | |
| ^‡^ C to T nucleotide transition rate held constant and other nucleotide transition rates estimated relative to it in the General Time Reversible (GTR) +Γ_4_ substitution model | | | | |

Table S6 – Prior distributions and posterior estimates of phylogenetic and epidemic parameters for the polymerase subunit PB2 nucleotide sequences isolated from wild birds and typed based on Eurasian source H5N8 and Eurasian/North American reassortant H5N2 isolated prior to 1 February 2015 during the highly pathogenic avian influenza virus outbreak on North America, 2014 – 2015. Posterior estimates obtained from combining 4 independent Markov-chain Monte Carlo runs of 10 million iterations after discarding 10% burn-in and sampling every 1,000^th^ iteration.

| Parameter* | Prior Distribution | Posterior Estimate mean [95% HPD] | Effective sample size^†^ | Gelman-Rubin potential scale reduction factor 95% upper bound |
| --- | --- | --- | --- | --- |
| **Epidemiological** |  |  |  |  |
| R_0_ H5N2 | lnNorm(0, 1.5) | 2.95  [0.95, 5.793] | 2,852 | 1.01 |
| R_0_ H5N8 | lnNorm(0, 1.5) | 2.68  [0.92, 5.13] | 3,564 | 1.01 |
| Lineage death rate (year^-1^) | lnNorm(2.5, 1) | 9.25  [2.41, 17.61] | 2,032 | 1.01 |
| H5N2 to H5N8 virus migration rate ( year^-1^) | Exp(1) | 0.65  [3.41E-4, 1.89] | 3,727 | 1.00 |
| H5N8 to H5N2 virus migration rate (year^-1^) | Exp (1) | 0.63  [7.77E-5, 1.75] | 3,590 | 1.01 |
| Sampling proportion H5N2 | Beta(1, 1) | 0.70  [0.28 1.0] | 3,487 | 1.00 |
| Sampling proportion H5N8 | Beta(1, 1) | 0.53  [0.11, 0.99] | 1,780 | 1.01 |
| **Phylogenetic** |  |  |  |  |
| Proportion invariant nucleotide sites | Unif(0, 1) | 0.29  [7.76E-4, 0.68] | 664 | 1.01 |
| uncorrelated relaxed lognormal molecular clock rate mean | lnNorm(-5.75, 3.5) | 7.96E-3  [3.69E-3, 0.01] | 1,251 | 1.03 |
| uncorrelated relaxed lognormal molecular clock rate standard deviation | Gamma(0.540, 0.385) | 0.18  [7.14E-9, 0.60] | 6,340 | 1.00 |
| A to C nucleotide transition rate | Gamma(0.05, 10) | 0.05  [2.06E-3, 0.16] | 779 | 1.01 |
| A to G nucleotide transition rate | Gamma(0.05, 10) | 1.08  [0.49, 1.86] | 1,408 | 1.01 |
| A to T nucleotide transition rate | Gamma(0.05, 10) | 3.67E-3  [3.24E-25, 0.02] | 220 | 1.00 |
| C to G nucleotide transition rate | Gamma(0.05, 10) | 2.41E-3  [1.62E-22, 0.01] | 129 | 1.07 |
| G to T nucleotide transition rate | Gamma(0.05, 10) | 0.23  [0.03, 0.50] | 1,517 | 1.01 |
| C to T nucleotide transition rate | 1^‡^ |  |  |  |
| * Parameters used to specified in birth-death multitype model template implemented in program Beauti v2.4.3 (Bouckaert et al., 2014; Kuhnert et al., 2016) | | | | |
| ^†^ Effective sample size calculated in program Tracer v.1.6.0 (Rambaut et al., 2014) | | | | |
| ^‡^ C to T nucleotide transition rate held constant and other nucleotide transition rates estimated relative to it in the General Time Reversible (GTR) +Γ_4_ substitution model | | | | |

Table S7 – Prior distributions and posterior estimates of phylogenetic and epidemic parameters for the matrix protein (M) nucleotide sequences isolated from wild birds and typed based on Eurasian source H5N8 and Eurasian/North American reassortant H5N2 isolated prior to 1 February 2015 during the highly pathogenic avian influenza virus outbreak on North America, 2014 – 2015. Posterior estimates obtained from combining 4 independent Markov-chain Monte Carlo runs of 10 million iterations after discarding 10% burn-in and sampling every 1,000^th^ iteration.

| Parameter* | Prior Distribution | Posterior Estimate mean [95% HPD] | Effective sample size^†^ | Gelman-Rubin potential scale reduction factor 95% upper bound |
| --- | --- | --- | --- | --- |
| **Epidemiological** |  |  |  |  |
| R_0_ H5N2 | lnNorm(0, 1.5) | 1.93  [0.81, 3.36] | 3,550 | 1.01 |
| R_0_ H5N8 | lnNorm(0, 1.5) | 1.60  [0.56, 2.83] | 4,525 | 1.00 |
| Lineage death rate (year^-1^) | lnNorm(2.5, 1) | 17.85  [5.56, 32.1] | 1,919 | 1.00 |
| H5N2 to H5N8 virus migration rate ( year^-1^) | Exp (1) | 1.76  [2.57E-4, 4.0] | 2,343 | 1.00 |
| H5N8 to H5N2 virus migration rate (year^-1^) | Exp (1) | 0.80  [7.25E-5, 2.23] | 4,279 | 1.00 |
| Sampling proportion H5N2 | Beta(1, 1) | 0.75  [0.36, 1.0] | 4,137 | 1.00 |
| Sampling proportion H5N8 | Beta(1, 1) | 0.65  [0.19, 1.0] | 1.109 | 1.01 |
| **Phylogenetic** |  |  |  |  |
| Proportion invariant nucleotide sites | Unif(0, 1) | 0.37  [2.02-45 0.77] | 796 | 1.02 |
| uncorrelated relaxed lognormal molecular clock rate mean | lnNorm(-6.1, 3.5) | 0.01  [4.37E-3, 0.02] | 1,920 | 1.00 |
| uncorrelated relaxed lognormal molecular clock rate standard deviation | Gamma(0.540, 0.385) | 0.15  [6.66E-9, 0.55] | 12,297 | 1.00 |
| A to C nucleotide transition rate | Gamma(0.05, 10) | 0.01  [1.52E-17, 0.04] | 235 | 1.24 |
| A to G nucleotide transition rate | Gamma(0.05, 10) | 0.82  [0.15, 1.67] | 1,204 | 1.03 |
| A to T nucleotide transition rate | Gamma(0.05, 10) | 7.92E-3  [2.48E-28, 0.04] | 192 | 1.06 |
| C to G nucleotide transition rate | Gamma(0.05, 10) | 4.08E-3  [3.16E-32, 0.02] | 177 | 1.03 |
| G to T nucleotide transition rate | Gamma(0.05, 10) | 0.26  [3.43E-3, 0.66] | 715 | 1.01 |
| C to T nucleotide transition rate | 1^‡^ |  |  |  |
| * Parameters used to specified in birth-death multitype model template implemented in program Beauti v2.4.3 (Bouckaert et al., 2014; Kuhnert et al., 2016) | | | | |
| ^†^ Effective sample size calculated in program Tracer v.1.6.0 (Rambaut et al., 2014) | | | | |
| ^‡^ C to T nucleotide transition rate held constant and other nucleotide transition rates estimated relative to it in the General Time Reversible (GTR) +Γ_4_ substitution model | | | | |

Figure S1- Time-rooted maximum clade credibility phylogenetic tree of the polymerase subunit (PB2) segment of clade 2.3.4.4 highly pathogenic avian influenza viruses isolated from wild birds and poultry during the 2014-2015 outbreak in North America. Eurasian (EA) source H5N8 subtype re-assorted with North American (NA) low-pathogenic viruses to form EA/NA H5N2 and EA/NA H5N1 subtypes. Tree tip circle colors represent host types of the isolates and pie charts on internal nodes display the posterior probability of host type of common ancestor viruses at majority rule common ancestors (posterior node probability >0.5). Bars represent uncertainty (95% highest posterior density intervals) of the divergence time of majority rule common ancestors.


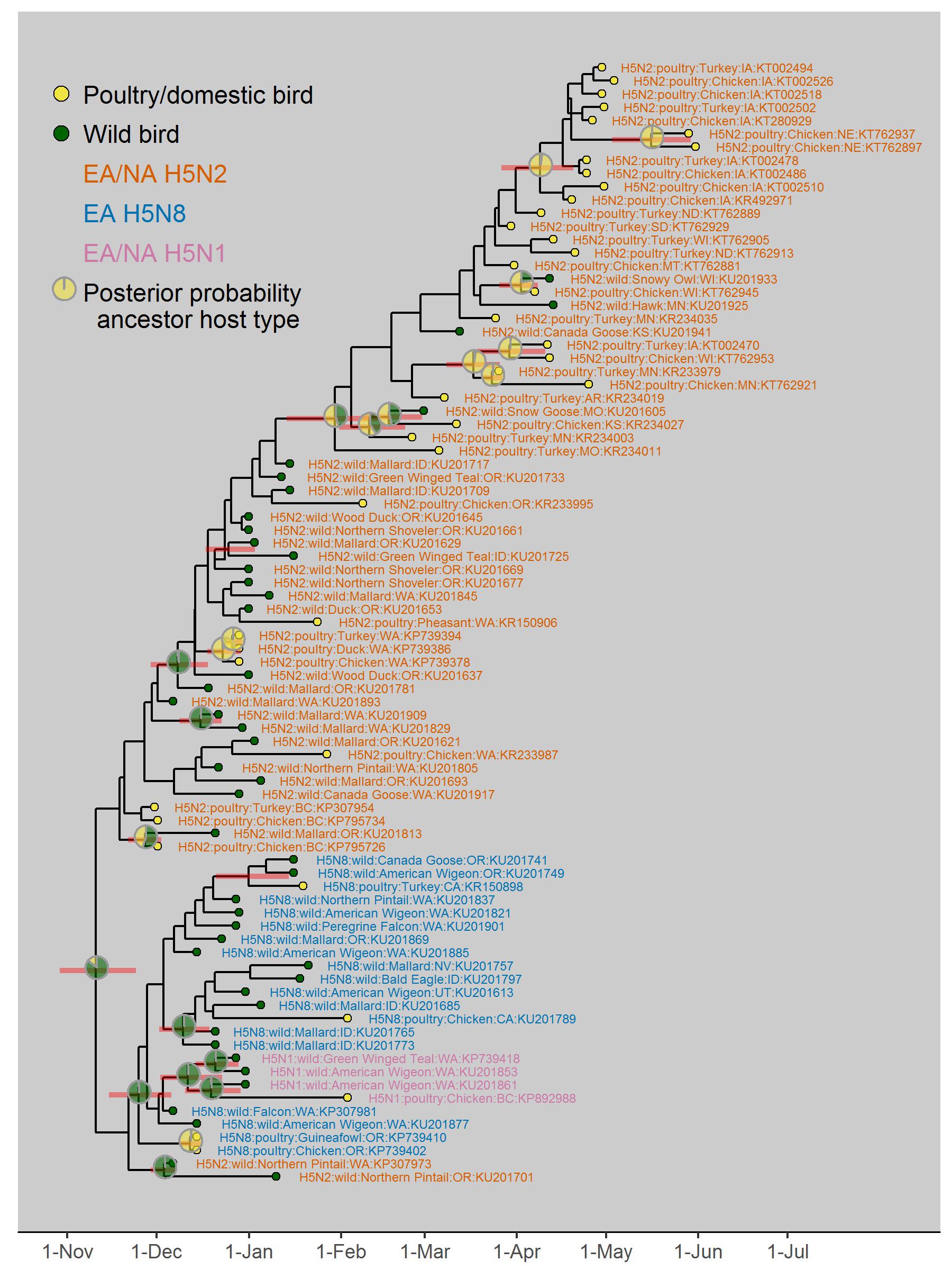


Figure S2- Time-rooted maximum clade credibility phylogenetic tree of the matrix (M) gene segment of clade 2.3.4.4 highly pathogenic avian influenza viruses isolated from wild birds and poultry during the 2014-2015 outbreak in North America. Eurasian (EA) source H5N8 subtype re-assorted with North American (NA) low-pathogenic viruses to form EA/NA H5N2 and EA/NA H5N1 subtypes. Tree tip circle colors represent host types of the isolates and pie charts on internal nodes display the posterior probability of host type of common ancestor viruses at majority rule common ancestors (posterior node probability >0.5). Bars represent uncertainty (95% highest posterior density intervals) of the divergence time of majority rule common ancestors.


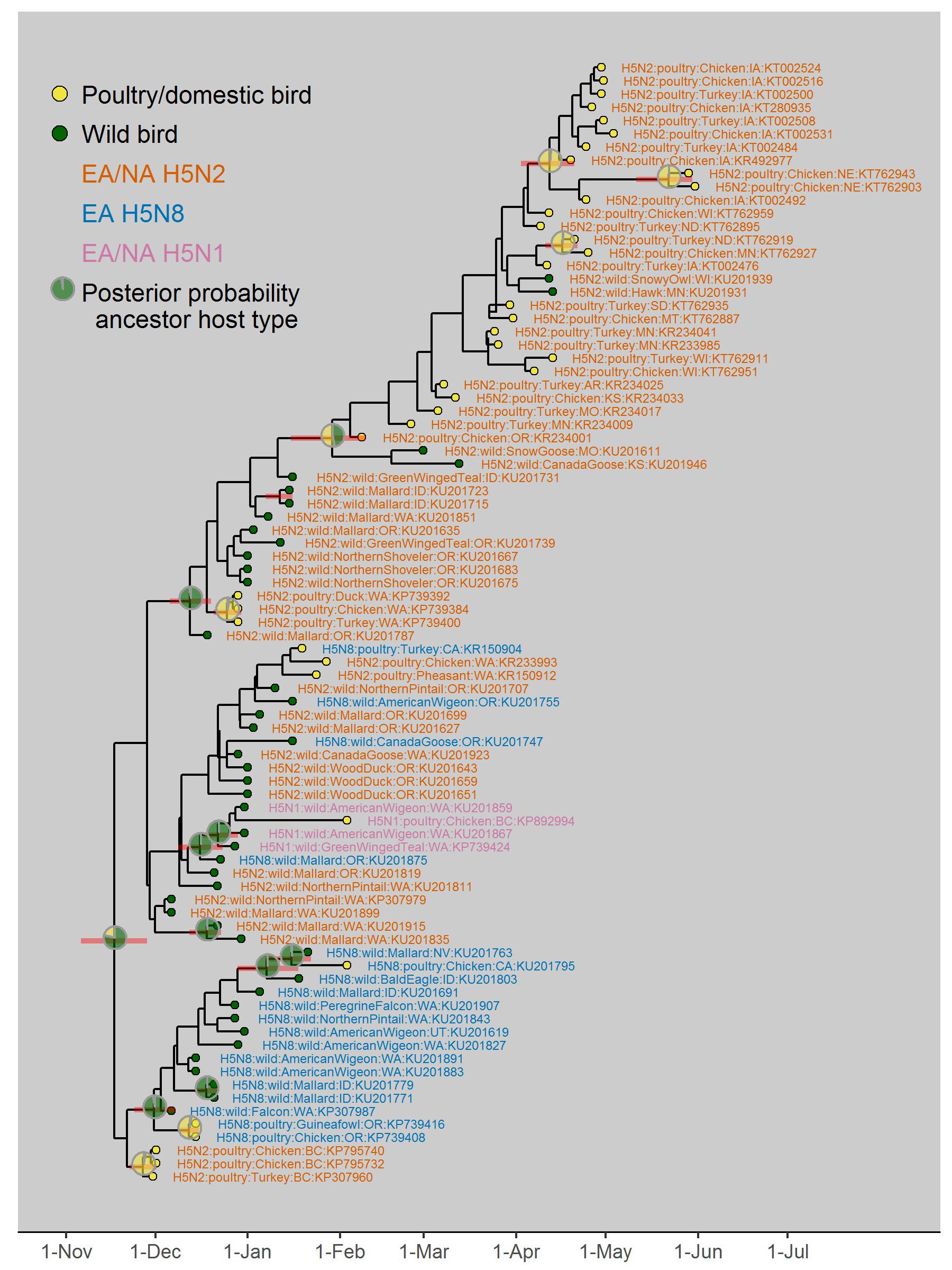

Supplement: Supplementary file 1 [file EVA-11-547-s001.docx]
